# Overcoming multidimensional immunotherapy resistance in PDAC: from microenvironment to clinic

**DOI:** 10.3389/fimmu.2026.1782960

**Published:** 2026-03-18

**Authors:** Jin Yan, Huiyi Ou, Shuai Wang, Kaizhou Jin, Xianjun Yu, Weiding Wu

**Affiliations:** 1 Department of Pancreatic Surgery, Fudan University Shanghai Cancer Center, Shanghai, China; 2 Department of Oncology, Shanghai Medical College, Fudan University, Shanghai, China; 3 Shanghai Pancreatic Cancer Institute, Shanghai, China; 4 Shanghai Key Laboratory of Precision Medicine for Pancreatic Cancer, Shanghai, China; 5 Pancreatic Cancer Institute, Fudan University, Shanghai, China

**Keywords:** dendritic cell (DC) immunotherapy, immunotherapy resistance, innate lymphoid cells, neoantigen vaccines, pancreatic ductal adenocarcinoma (PDAC), tumor immune microenvironment (TME)

## Abstract

Pancreatic ductal adenocarcinoma (PDAC) remains one of the deadliest cancers, with immunotherapy yielding <10% objective response rates (ORR) due to its profoundly immunosuppressive tumor microenvironment (TME). This review integrates preclinical and clinical evidence (2018-2026) to dissect how stromal desmoplasia, myeloid dominance, T-cell exclusion, and impaired antigen presentation converge to form an immune-privileged niche. Key resistance pathways, including cGAS-STING, Hedgehog, and NF-κB, are discussed alongside emerging strategies such as CAR-T cells, mRNA neoantigen vaccines, STING agonists, CD39/CD73 blockade, and cDC1-based vaccines. Despite incremental progress, durable responses remain rare, emphasizing that single-target interventions are insufficient. We propose a “3D+R” framework, De-desmoplasia, De-adenosine, *De-novo* antigen, and Rational sequencing, to guide multidimensional, biomarker-driven immunotherapy design. Approaches such as timed cDC1 vaccination, patient-tuned STING agonism, and metabolic checkpoint inhibition exemplify how PDAC’s immune-desert phenotype may be reshaped toward an immune-reactive state. Conceptualizing PDAC as a dynamic immune ecosystem rather than a mutation-driven entity may ultimately transform sporadic responses into durable and predictable clinical benefit.

## 1 Introduction

PDAC is the most common form of pancreatic cancer, accounting for over 90% of cases (1, 2). Despite surgical resection, chemotherapy, and targeted therapy, outcomes remain poor due to high recurrence rates and limited therapeutic durability (3–5).

A hallmark of PDAC is a desmoplastic and immunosuppressive TME enriched with cancer-associated fibroblasts (CAFs), extracellular matrix components, and immunosuppressive immune cells such as regulatory T cells (Tregs), myeloid-derived suppressor cells (MDSCs), and tumor-associated macrophages (TAMs), particularly the M2-like phenotype (6). This environment not only impedes cytotoxic T cell infiltration but also actively promotes T cell exhaustion and immune evasion through mechanisms including checkpoint ligand expression, metabolic competition, and secretion of inhibitory cytokines such as TGFβ and IL-10. Moreover, antigen-presenting cells (APCs), like dendritic cells (DCs) in PDAC are often immature or dysfunctional, further reducing T cell priming (7). Collectively, these features indicate that immunotherapy resistance in PDAC is not attributable to a single immune defect. Instead, it emerges from coordinated stromal, metabolic, antigenic, and temporal barriers. Accumulating evidence suggests that these barriers converge on a distorted immune balance, characterized by regulatory dominance over effector T-cell states. Accordingly, balance-oriented metrics such as the effector-to-regulatory T-cell ratio have been proposed as clinically interpretable indicators of immune dysfunction and potential tools for biomarker-driven stratification and therapeutic sequencing (8). Extensive desmoplasia physically and functionally excludes effector lymphocytes; adenosine-rich metabolic checkpoints suppress cytotoxic immunity; impaired antigen presentation limits effective T-cell priming; and inappropriate sequencing of immunotherapies further blunts clinical efficacy. As a result, even rational combinations frequently fail to generate durable responses.

**Figure 1:**
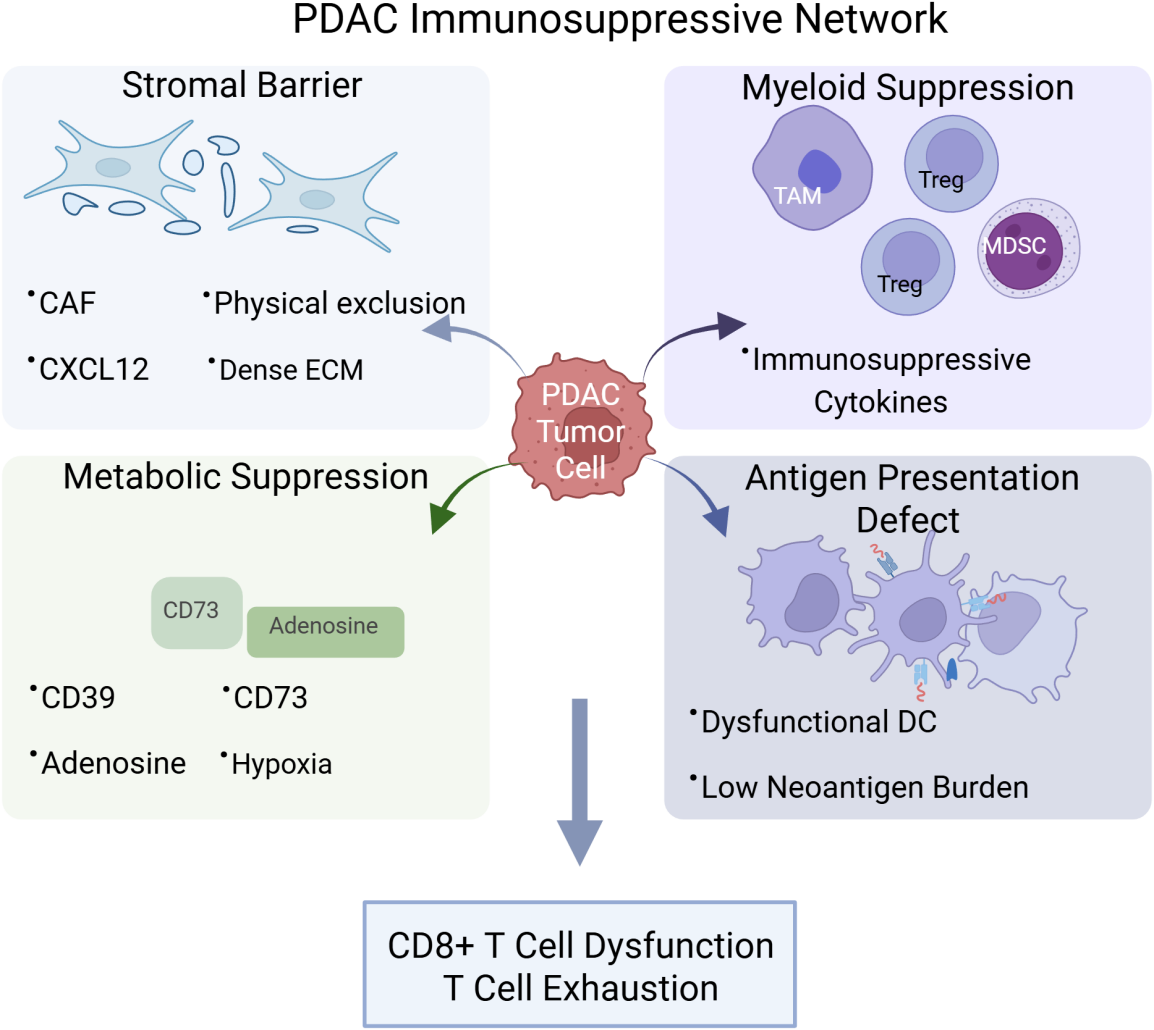
Multidimensional immune resistance in PDAC. PDAC immune evasion arises from coordinated stromal exclusion, metabolic suppression, myeloid dominance, and impaired antigen presentation. These interdependent barriers converge to drive CD8^+^ T-cell dysfunction and resistance to immune checkpoint blockade.

To conceptualize these interdependent resistance mechanisms and to guide next-generation therapeutic design, we propose a “3D+R” framework, De-desmoplasia, De-adenosine, *De-novo* antigen, and Rational sequencing. The multidimensional resistance landscape underlying this framework is illustrated in **Figure 1**. This framework views PDAC not as a static, mutation-driven entity, but as a dynamic immune ecosystem in which therapeutic success depends on dismantling multiple non-redundant barriers in a temporally coordinated and biomarker-informed manner. Representative preclinical and clinical strategies corresponding to each 3D+R dimension are summarized in **Table 1**.

**Table 1:** Conceptual integration of representative PDAC immunotherapy strategies within the 3D+R framework.

Study/Strategy	Intervention	Primary immune barrier addressed	3D+R dimension	Proposed biomarker/stratification	Key outcome or limitation
CSF1R-targeting	anti-CSF1R ± ICB ± chemotherapy	Myeloid suppression	Rational sequencing	Not defined	Limited T-cell recovery
CD73 blockade	anti-CD73 + anti-PD-1	Adenosine suppression	De-adenosine	CD73 expression	Limited efficacy
CD73 + chemotherapy	anti-CD73 + chemotherapy	Adenosine suppression	De-adenosine	Not defined	Combination dependency
STING activation	STING agonist combinations	Innate sensing defect	*De-novo* antigen	STING competence	Context dependence
GVAX vaccination	GVAX combinations	Antigen presentation deficit	*De-novo* antigen	TLS formation	No survival benefit
AXL inhibition	AXL inhibitor + ICB	Stromal exclusion	De-desmoplasia	AXL expression	Insufficient activation
TGFβ inhibition	TGFβ trap combinations	CAF suppression	De-desmoplasia	TGFβ activity	Biomarker dependence
Multimodal sequencing	Chemotherapy + ICB + radiotherapy	Immune exclusion	Rational sequencing	Immune phenotype	Timing dependence
cDC1 vaccination	cDC1 vaccine + ICB	Antigen presentation deficit	*De-novo* antigen	cDC1 abundance	Preclinical efficacy only
Dual immune targeting	Checkpoint + myeloid co-targeting	T-cell exhaustion	Rational sequencing	Treg/CD8^+^ ratio	Multi-barrier requirement

## 2 T cell-based therapeutic strategies

The TME of PDAC profoundly restricts effective T-cell responses. Although subsets like mismatch repair-deficient (dMMR) PDAC (~1-2% of cases) show durable responses to PD-1 blockade owing to their high neoantigen load, the majority of PDACs are resistant to checkpoint inhibitors due to low T-cell infiltration, metabolic dysfunction, and multiple inhibitory receptor expressions on tumor-infiltrating lymphocytes (9). To alleviate this state of suppression, researchers have proposed a variety of methods to enhance T-cell immune function from multiple perspectives.

### 2.1 CAR-T therapy

Chimeric antigen receptor T-cell (CAR-T) therapy employs genetically engineered T cells to target cancer cells (10). This approach may enhance CD8^+^ T-cell infiltration within the pancreatic TME and sensitize tumors to immune checkpoint blockade (ICB). However, CAR-T therapy in PDAC remains exploratory and lacks definitive clinical validation (11). Several challenges remain unresolved, including antigen loss due to genetic alterations, functional impairment of CAR-T cells within the immunosuppressive PDAC microenvironment, and cytokine release-associated toxicities such as fever, hypotension, and organ dysfunction (12). To address these limitations, engineering CAR-T cells to express cytokines such as IL-17 or CCL19 has been shown to enhance intratumoral infiltration and persistence (13). In addition to tumor-derived neoantigens, the abundant PDAC stroma provides a reservoir of alternative target antigens (14). Notably, cellular immunotherapy is evolving beyond conventional CAR-T constructs toward next-generation engineering strategies designed to enhance persistence, controllability, and functional fitness within hostile solid-TMEs. Emerging RNA-based regulatory concepts, including circular RNA-enabled approaches, exemplify how CAR-T design is being modernized to address limitations that are particularly pronounced in PDAC (15).

### 2.2 Vaccines for boosting T cells

#### 2.2.1 mRNA-based vaccines

Conventional vaccination platforms induce limited immunity in PDAC, mandating antigen-tailored and microenvironment-aware designs. PDAC is inherently characterized as a low-mutated, cold tumor, with proteins associated with neoantigens that are virtually not present in healthy tissues (16), consequently impeding dense T cell infiltration. Luis A. Rojas and colleagues have derived and synthesized a personalized mRNA neoantigen vaccine, autogene cevumeran, in real-time from PDAC tumors that have been surgically excised. Individuals who received this customized vaccine demonstrated a markedly prolonged median recurrence-free survival. Furthermore, this vaccine has been shown to amplify CD8^+^ T cells that are specific to neoantigens, exhibit functionality, and persist over time, which is of considerable significance for the immunotherapy of PDAC, a tumor type that is not typically inflammatory (17). This method of personalized treatment targets the tumor’s unique mutations to provoke a specific immune response. Ongoing advances in RNA technology may further optimize vaccine efficacy and manufacturing quality, potentially improving postoperative outcomes in selected PDAC patients.

#### 2.2.2 Granulocyte-macrophage colony-stimulating factor-secreting allogeneic PDAC cell vaccine

Granulocyte-macrophage colony-stimulating factor-secreting allogeneic PDAC cell vaccine (GVAX) is a GM-CSF-secreting whole-cell vaccine that can activate T-cell antitumor responses, induce the formation of tertiary lymphoid aggregates (LA), and upregulate PD-L1 within LA (18). For the robust immunosuppressive environment of PDAC, the efficacy of GVAX alone is extremely limited, and even when combined with ipilimumab, it results in only marginal improvements in overall survival (OS) for PDAC patients (19). CRS-207 is an attenuated Listeria monocytogenes vaccine targeting mesothelin and has demonstrated antitumor activity in various cancers. In a Phase 2b study, the combination of cyclophosphamide, GVAX, and CRS-207 showed potential survival benefits in early trials compared to cyclophosphamide monotherapy, but OS was not significantly prolonged (20). Thus, it is evident that GVAX or CRS-207 alone is insufficient to overcome the immunosuppressive microenvironment of PDAC.

#### 2.2.3 SVN-2B peptide vaccine

SVN-2B peptide vaccine is a tumor-specific immunotherapy based on the survivin protein, an anti-apoptotic protein whose 2B variant encodes peptides that can induce a robust immune response. A study analyzed the immune status of PDAC lesions post-vaccination through autopsy and revealed that the SVN-2B peptide vaccine could induce the infiltration of cytotoxic T lymphocytes (CTLs), although the expression of PD-L1 on tumor cells likely inhibited the antitumor effects of CTLs (21), Combining SVN-2B with ICBs may represent an ideal therapy with both good efficacy and fewer severe side effects. In another Phase II clinical study, interferon-β was used as an adjuvant, significantly increasing the infiltration of SVN-2B-specific CTLs and prolonging survival post-progression (SPP). Although clinical translation studies on the SVN-2B vaccine are currently limited, its potential for early or adjuvant therapy is evident (22).

#### 2.2.4 GV1001

GV1001 is a peptide vaccine targeting telomerase, designed to activate T-cell immune responses to attack tumor cells expressing telomerase. Telomerase is highly expressed in most PDAC, making it a potential therapeutic target (23). However, GV1001 failed to extend OS versus gemcitabine/capecitabine alone (24), *Post-hoc* immunomonitoring revealed that only approximately 20% of recipients mounted detectable telomerase-specific T-cell responses, and preceding chemotherapy-induced lymphopenia further blunted vaccine immunogenICBty, explaining the lack of survival benefit in unselected patients (25).

#### 2.2.5 KIF20A

The KIF20A peptide vaccine was developed based on the identification of KIF20A-66. The KIF20A-66 peptide has been identified as a novel HLA-A24-restricted tumor-associated antigen with significant trans-activating properties in PDAC. Patients vaccinated with this peptide demonstrated better clinical outcomes (26), and those receiving a peptide vaccine containing KIF20A as adjuvant therapy post-surgery exhibited KIF20A-specific CD8^+^ cell responses (27), suggesting potential benefit.

### 2.3 Signaling pathway related to T cells

#### 2.3.1 cGAS-STING

The cGAS-STING signaling pathway is a central component of innate immune sensing, responsible for detecting aberrant cytosolic double-stranded DNA and initiating immune activation. Upon recognizing cytoplasmic DNA, cyclic GMP-AMP synthase (cGAS) catalyzes the production of the second messenger cGAMP, which subsequently binds to and activates STING (stimulator of interferon genes), leading to downstream signaling that induces type I interferons (IFN-I) and pro-inflammatory cytokines (28). Unlike normal cells, cancer cells frequently harbor cytosolic double-stranded DNA, rendering this pathway particularly relevant in tumor immunity.

In PDAC, the cGAS-STING axis exerts context-dependent effects. STING is expressed in approximately 90% of PDAC cases, and co-expression of cGAS and STING in tumor cells is associated with improved survival and increased infiltration of cytotoxic CD8^+^ T cells (29). Beyond IFN secretion, STING activation can reduce immunosuppressive cell populations while promoting T-cell and NK-cell infiltration, thereby supporting durable antitumor immune memory. Consistently, preclinical studies demonstrate that pharmacological STING agonists can directly activate this pathway, enhancing antitumor immunity independently of tumor-derived DNA release, and have been incorporated into PDAC vaccine strategies (30).

However, excessive or inappropriate STING activation may paradoxically promote tumor progression when tumors co-opt this pathway for oncogenic signaling and immune suppression (28). STING-targeted therapies are unlikely to benefit all PDAC patients. Careful patient selection, optimized dosing, and rational combination strategies are therefore required. Additional experimental and clinical studies are required to define the therapeutic window, toxicity profile, and combinatorial potential of STING agonists.

#### 2.3.2 Hedgehog

Aberrant activation of the Hedgehog (HH) signaling pathway is associated with the onset and progression of PDAC (31). In PDAC, HH signaling exhibits cell-type-specific activity within the stroma, particularly in myofibroblastic cancer-associated fibroblasts (myCAFs) and inflammatory CAFs (iCAFs). Pharmacological inhibition of HH signaling reduces myCAF abundance while increasing iCAFs; however, this stromal remodeling is accompanied by immunological trade-offs. HH inhibition has been shown to decrease CD8^+^ T-cell infiltration and increase regulatory T cells (CD4^+^CD25^+^), thereby inducing a degree of immune suppression (32).

Clinically, FDA-approved HH inhibitors have failed to improve outcomes in metastatic PDAC, with acquired resistance and dose-limiting toxicities, including grade ≥3 hyponatraemia, diarrhea, and fatigue, frequently necessitating treatment modification (33). These findings highlight the dual and context-dependent role of HH signaling in PDAC, where pathway inhibition can both disrupt tumor-promoting stroma and inadvertently impair antitumor immunity. Consequently, HH-targeted strategies require careful patient selection and rational combination with other immunotherapies to mitigate immunosuppressive effects and maximize therapeutic benefit (32).

#### 2.3.3 NF-κB signaling pathway

The NF-κB signaling pathway plays a pivotal role in coordinating inflammatory responses and innate immune signaling in PDAC. Interleukin-1 receptor-associated kinase 4 (IRAK4) is a key upstream regulator of this pathway and has emerged as a potential therapeutic target (34). Experimental studies demonstrate that IRAK4-dependent NF-κB activation promotes PDAC cell survival and stromal fibrosis. Genetic deletion or pharmacological inhibition of IRAK4 abrogates NF-κB activity, reduces the expression of immunosuppressive factors, checkpoint ligands, and hyaluronan synthase 2 (HAS2), and markedly diminishes stromal fibrosis.

In KPC mouse models, IRAK4 inhibition reprograms myeloid and fibroblastic cells toward an acute inflammatory phenotype, resulting in enhanced infiltration and activity of CD4^+^ and CD8^+^ T cells. Importantly, combining IRAK4 inhibitors with immune checkpoint blockade significantly prolongs survival in these models, providing a strong mechanistic rationale for combinatorial immunotherapy (35). Collectively, targeting the IRAK4-NF-κB axis represents a strategy to enhance T-cell responsiveness and improve combinatorial immunotherapy efficacy. Within the 3D+R framework, IRAK4-NF-κB pathway modulation primarily supports the “De-desmoplasia” axis by influencing stromal–immune crosstalk.

### 2.4 Fostering T cells at the genetic level

Gene engineering techniques have identified a multitude of therapeutic targets for PDAC. Among these, the Clustered Regularly Interspaced Short Palindromic Repeats (CRISPR) technology has been extensively utilized in the immunotherapy of PDAC. The CRISPR/Cas9 system can be employed to disrupt the PD-1 gene in T cells, thereby enhancing their antitumor activity and improving their functional capacity to more effectively combat PDAC cells (36). Additionally, leveraging CRISPR technology, Liu et al. constructed a mouse PDAC cell model with the gene for quinoid dihydropteridine reductase (QDPR), a key enzyme in biopterin metabolism, knocked out. The deficiency of QDPR leads to the accumulation of dihydrobiopterin (BH2), reducing the ratio of tetrahydrobiopterin (BH4) to BH2, and ultimately resulting in resistance to ICB, suggesting that supplementation of BH4 may enhance the efficacy of ICB in PDAC patients (37). Nevertheless, issues such as off-target editing and low delivery efficiency associated with CRISPR technology need to be resolved to facilitate broader application of the CRISPR/Cas9 system in future clinical treatments.

### 2.5 Metabolic checkpoints - adenosine axis

Beyond cell vaccines, the ATP-adenosine axis is a key metabolic immune checkpoint in PDAC, where CD39 and CD73 convert extracellular ATP into immunosuppressive adenosine, inhibiting T and natural killer (NK) cells (38). Increasing evidence positions CD39 (ENTPD1) as a central gatekeeper of adenosine-driven immune suppression rather than a passive upstream enzyme, with emerging translational relevance not only as a therapeutic target but also as a biomarker of metabolically suppressed immune states within the PDAC TME (39, 40). CD73 catalyses AMP→adenosine, thereby amplifying immunosuppression within the tumor bed. Upstream, the first-in-class anti-CD39 IgG4 mAb TTX-030 blocks ATP hydrolysis; a phase II trial (NCT06119217) is evaluating gemcitabine/nab-paclitaxel ± TTX-030 ± budigalimab, with acceptable early safety but pending efficacy (41). Conceptually, CD39 blockade alone may be insufficient, supporting its integration within biomarker-guided combination strategies (39). Downstream, the A_2_aR antagonist ciforadenant plus pembrolizumab achieved only 5% ORR in a phase I pancreatic cohort, leading to deprioritized development (42). These data highlight the need for rational sequencing or triple combinations with chemotherapy, ICBs, and stroma-modifying agents. Within the 3D+R framework, these T-cell-oriented and metabolic interventions align with the “*De-novo* antigen” and “De-adenosine” dimensions, underscoring that effective immune activation requires simultaneous relief of stromal and metabolic constraints.

## 3 Antigen-presenting cell-directed immunotherapies

APCs, particularly DCs, play a pivotal role in initiating antitumor immune responses by capturing, processing, and presenting tumor antigens to T cells. DCs are professional APCs that link the innate and adaptive immune systems (43). Upon antigen uptake, activated DCs process antigenic peptides and present them in association with major histocompatibility complex (MHC) molecules to naive T lymphocytes, triggering specific immune responses (44). Different subtypes have distinct functional emphases (45). DCs are categorized into several subtypes, with conventional DCs (cDCs) being the most crucial for antitumor immunity. cDC1 and cDC2 are the two principal subsets, each with distinct immunological functions. cDC1s are specialized in cross-presenting antigens to CD8^+^ T cells and promoting CTL responses, whereas cDC2s are more adept at activating CD4^+^ T helper cells, particularly Th2 and Th17 subsets (46). In healthy tissues, both subsets maintain immune surveillance, but in PDAC, DCs, especially cDC1s, are markedly reduced, contributing to immune evasion (47). The lack of DCs in the PDAC microenvironment impairs T cell priming and facilitates tumor progression.

### 3.1 Agonist of dendritic cells

Restoring cDC1 presence using Flt3L, a DC growth factor (48), reinvigorates CD8^+^ T cells and sensitizes tumors to ICB. Vaccination with cDC1s has been shown to synergize with ICB and establish long-term immune memory in preclinical PDAC models, underscoring their non-redundant role in antitumor immunity (49). As a specific example, Samarth Hegde and colleagues demonstrated that in pancreatic cancer-bearing KPC mice, cDC1s significantly enhanced CD8^+^ T cell and Th1 responses, while suppressing IL-17 production by Th17 cells. Their study utilized Flt3L to mobilize DCs into early PDAC lesions, effectively reversing fibroinflammatory responses within the TME (50). These results not only support the central role of cDC1s in regulating T cell polarization but also provide a rationale for incorporating DC-targeting strategies into PDAC immunotherapy.

### 3.2 Synthesis of dendritic cells *in vitro*

A DC-based adjuvant immunotherapy for the prevention of pancreatic cancer recurrence has been proposed: by inducing the differentiation of patient-derived monocytes into DCs *in vitro* and exposing them to tumor lysates to “mature” the DCs, followed by re-infusion into patients who have undergone surgical resection of pancreatic cancer lesions, the recurrence-free survival rate within two years post-surgery can reach 60% (51). Additionally, research has demonstrated that the use of DC vaccines following non-thermal ablation significantly improves the survival rate in PDAC model mice (52).

### 3.3 Dendritic cell-related vaccines

MUC1 is a highly glycosylated transmembrane protein that is often aberrantly overexpressed in PDAC, thus becoming a target for PDAC vaccine development (51). The MUC1-Vax-DC vaccine can simultaneously activate cellular and humoral immune responses, significantly inhibiting the growth of MUC1+ and PD-L1+ tumor cells and prolonging the survival of model mice (52). A study designed a non-natural MUC1cope glyptide homogeneous cancer vaccine with higher immunogenicity, which significantly reduced tumor volume and prolonged survival in mouse models when used alone (53).

### 3.4 Enhancing APCs through genetic engineering

Another team discovered that the deletion of the galectin-3 (GAL3) gene in PDAC model mice significantly inhibited the progression of spontaneous pancreatic tumors and extended the survival of the mice. Single-cell analysis revealed that the absence of GAL3 led to a compensatory increase in the chemokine CXCL12 and a notable elevation in the number of antitumor myeloid cell subsets expressing high levels of MHC II, which helps to activate the antitumor immune response (54).

## 4 Natural killer cell-based therapeutic strategies

NK cells are innate cytotoxic lymphocytes critical for tumor immune surveillance. However, in PDAC, NK cells are profoundly impaired both in number and function. Studies have shown that infiltrating NK cells are extremely scarce in PDAC tumor tissue due to defective localization mechanisms. Specifically, NK cells from PDAC patients exhibit reduced chemokine receptor expression (e.g., CXCR2), which prevents their migration into tumor sites. Moreover, these NK cells downregulate activating receptors such as NKG2D and DNAM-1, resulting in impaired cytotoxicity and facilitating tumor immune escape (55). Beyond cytotoxic deficiency, PDAC-infiltrating NK cells may acquire regulatory features, including IL-10 secretion and immune-suppressive profiles. This regulatory switch marks NK dysfunction as a cardinal feature of PDAC immune escape (56). To overcome these limitations, several innovative NK cell-based therapies are under development. A notable approach involves engineering off-the-shelf invariant natural killer T (iNKT) cells to express anti-PSCA chimeric antigen receptors (CAR) along with IL-15. These modified iNKT cells demonstrated potent antitumor activity and prolonged survival in preclinical PDAC model (57). Similarly, the use of CAR-transduced NK cells, while initially explored in hematologic malignancies, has opened a new avenue for solid tumors including PDAC (58). Another promising strategy is the generation of cytokine-induced memory-like NK cells, which display enhanced responsiveness and sustained cytotoxic function. These memory-like NK cells show superior activity against resistant tumors and may be adaptable to PDAC treatment (59). Collectively, these findings suggest that restoring NK localization and cytotoxic competence remains critical for therapeutic exploitation. Natural killer cell activity (NKA) in patients with PDAC closely associated with the postoperative recurrence rate. NKA decreased as cancer progressed (60), and those with low NKA values have a high risk of early recurrence and a poor prognosis (61). In addition, NK cell-based immunotherapy for PDAC has also successfully demonstrated positive therapeutic effects in preclinical metastatic PDAC models (62). NKA, reflected by cytotoxic degranulation capacity, correlates with disease progression and prognosis in PDAC patients. However, it may serve primarily as a prognostic biomarker rather than a direct therapeutic target, given its functional decline under tumor-induced immunosuppression (63). Consequently, NK-cell-directed strategies represent a complementary component of combination immunotherapy in PDAC. Within the 3D+R framework, NK-cell restoration aligns with the “*De-novo* antigen” and “Rational sequencing” dimensions.

## 5 B cell-based therapeutic strategies

B cells are highly infiltrated in PDAC, yet their roles are often overlooked. Within the TME of PDAC, B cells exhibit dual functions, capable of both tumor suppression and tumor promotion. Tertiary lymphoid structures (TLS) are newly formed ectopic lymphoid aggregates that can modulate immunity and enhance antitumor immunity within tumors. In tumors with mature TLS, the rate of B cell somatic hypermutation is increased, indicating that the formation of germinal centers within TLS occurs in the presence of high-quality tumor neoantigens, leading to enhanced humoral immunity and improved survival rates for PDAC patients (64). In the TME, B cells can differentiate into regulatory B cells (Bregs), which suppress antitumor immune responses through the secretion of anti-inflammatory cytokines, such as IL-10 and IL-35. Thus, targeting Bregs represents a previously under-appreciated therapeutic avenue in PDAC. The BTK signaling pathway and IL-18 are essential for the growth and development of Bregs, and their respective inhibitors can be used to inhibit tumor progression and metastasis (65). Studies have demonstrated that anti-IL-35 therapy not only reduces tumor growth but also enhances the efficacy of anti-PD-1 immunotherapy, indicating that IL-35 is a potential therapeutic target (66). Furthermore, research has confirmed the critical role of BCR signaling and PKD2 in regulating B cell function, providing new targets for the development of immunotherapeutic strategies against PDAC, particularly through the inhibition of BCR signaling and PKD2 to enhance antitumor immune responses (67). These observations further illustrate that immune balance restoration, including modulation of regulatory B-cell programs, must be integrated into the broader 3D+R strategy.

## 6 Mast cell-related studies

Compared to normal tissue, the infiltration of mast cells in PDAC tissue is significantly increased, primarily at the tumor periphery, which is associated with tumor progression. Mast cells stimulate the proliferation of cancer-associated fibroblasts (CAFs) through the secretion of cytokines and granules, thereby promoting the proliferation and invasion of PDAC cells. Studies have also shown that they may limit the efficacy of immunotherapy by suppressing immune responses: mast cells interact with dendritic cells to promote PDAC development and may inhibit T cell activity through the release of mediators such as histamine, thereby restricting immune responses (68). Collectively, mast-cell blockade warrants investigation as a strategy to alleviate PDAC immunosuppression. Researchers have observed that mast cell-deficient mice exhibit significantly prolonged survival, reduced tumor growth, and decreased fibrosis (69). Such findings suggest that mast-cell modulation may improve therapeutic responsiveness. Within the 3D+R framework, this approach aligns with the “De-desmoplasia” axis.

## 7 Other immune-related cells

**Figure 2:**
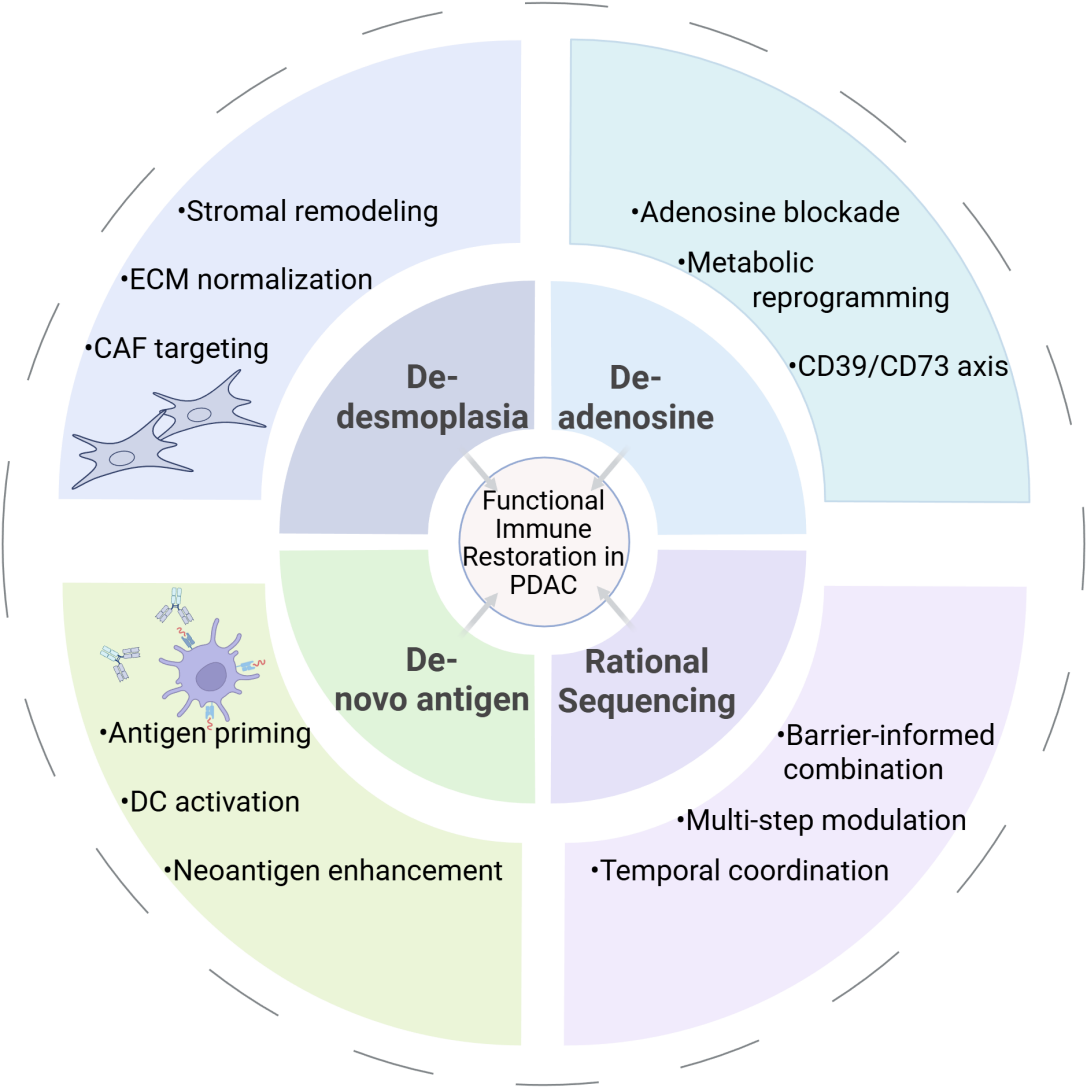
Cellular architecture of the PDAC immune microenvironment. Major stromal and immune cellular compartments within the PDAC TME and their functional interactions.

The team of Eileen S. Carpenter identified a unique intermediate subtype of tumor cell subset highly expressing keratin 17 (KRT17), interleukin 8 (CXCL8), and other cytokines. The KRT17^High^/CXCL8^+^ cells secrete CXCL8, which induces the migration of myeloid cells into the tumor. As myeloid cells here inhibit cytotoxic T-cell responses promoting carcinogenesis (70), this subtype of cells indirectly reinforces the immunosuppression in PDAC. During chemotherapy, patients with good therapeutic outcomes exhibit a decrease in CXCL8 levels in the blood, while the persistent presence of CXCL8 indicates poor prognosis (71). Despite the absence of empirical evidence supporting the efficacy of directly targeting KRT17^High^/CXCL8^+^ tumor cells in reversing the immunosuppression associated with PDAC and its potential to augment immunotherapeutic strategies, the prevalence of these cellular subsets within tumors could be employed as a criterion for patient stratification, thereby facilitating a more nuanced and precise therapeutic approach. A single-cell RNA sequencing study has identified a subpopulation of cancer-associated fibroblasts (CAFs) that express MHC II molecules, termed antigen-presenting CAFs (apCAFs). These apCAFs are capable of inducing the differentiation of naive CD4^+^ T cells into regulatory T cells (Tregs) in an antigen-specific manner. This process is independent of conventional co-stimulatory molecules and is instead mediated through the presentation of antigens by MHC II molecules (72). The apCAFs originate from mesothelial cells and acquire fibroblastic characteristics during the progression of PDAC via the IL-1 and TGFβ signaling pathways, thereby promoting the formation of the tumor stroma in PDAC. Future research may explore the potential of targeting the IL-1 and TGFβ signaling pathways to modulate the function of apCAFs, which could offer novel therapeutic strategies for the treatment of PDAC (73). Collectively, APC restoration strategies represent the core of the “*De-novo* antigen” dimension within the 3D+R model, as effective checkpoint therapy in PDAC fundamentally depends on competent dendritic-cell-mediated priming. A detailed cellular and molecular map of the PDAC immune microenvironment is shown in **Figure 2**.

## 8 Novel immune targets

In addition to conventional immune targets, emerging lymphocyte subsets such as mucosal-associated invariant T (MAIT) cells and innate lymphoid cells (ILCs) are gaining attention as potential next-generation immunotherapeutic targets in PDAC. MAIT cells have been detected within the PDAC TME and are often functionally impaired, expressing high levels of checkpoint receptors, and exhibiting reduced IFN-γ production. Recent preclinical models have shown that reactivating or engineering MAIT cells (e.g., CAR-MAIT approaches) enhances their cytotoxic potential and may avoid conventional CAR-T-associated toxicity (74). Meanwhile, ILC subsets, particularly ILC1s and ILC2s, have been increasingly characterized in human solid tumors, where their functional plasticity and cytokine-mediated crosstalk with other immune subsets offer a novel axis for modulating PDAC immunity (75).

## 9 Immunotherapy based on non-immune cell targets

### 9.1 Oncolytic virus therapy

OVs represent an emerging therapeutic approach in cancer treatment. Initially noted for their tumor-lytic effects in cancer patients over a century ago, these viruses have been meticulously selected and attenuated for therapeutic use. These viruses selectively infect tumor cells and can enhance antitumor immune responses (76). In 2017, research demonstrated that oncolytic viruses could modulate the metabolic characteristics of tumor cells, impairing energy production pathways through the reduction of glycolysis and the induction of metabolic stress. This leads to an increase in immunogenic cell death, rendering tumors more vulnerable to immunological destruction (77). In the context of PDAC, a cold tumor with immune suppressive properties and a limited presentation of neoantigen epitopes, oncolytic viruses are poised to overcome existing immunotherapeutic challenges. The versatility of oncolytic viruses is evident, with clinical trials underway for combinations such as Pelareorep with pembrolizumab chemotherapy. This synergistic strategy taps into the immune-activating potential of oncolytic viruses, complemented by the ICB-mediated immune enhancement afforded by ICBs (78).

### 9.2 Microenvironmental reprogramming

Gene engineering not only directly modifies the parenchymal cells of PDAC but also reprograms the PDAC immune microenvironment, shifting the immunosuppressive landscape toward an immune-permissive state. Inhibition of the eIF4G1 subunit of the eukaryotic initiation factor 4F (eIF4F) complex reduces the production of profibrotic cytokines and chemokines, effectively limiting tumor progression and extending survival (79).

## 10 Combination immunotherapy

ICB is currently one of the most frequently utilized immunotherapeutic agents. Programmed cell death protein 1 (PD-1)/programmed cell death ligand 1 (PD-L1) inhibitors and cytotoxic T-lymphocyte-associated protein 4 (CTLA-4) inhibitors are the most common types of ICB. ICB is widely used, yet its efficacy in PDAC remains limited (80). Although studies have indicated that ICB may have some palliative effect on microsatellite instability-high (MSI-H) PDAC, ORR for MSI-H PDAC patients treated solely with ICB is less than 20% (81). Furthermore, it is worth noting that the proportion of MSI-H PDAC cases is exceedingly rare (82). Nevertheless, the outcomes of their attempts have largely been disappointing: the ORR among subjects treated with the combination of a PD-L1 monoclonal antibody and a CTLA-4 monoclonal antibody was a mere 3.1% (83). Even with the employment of dual ICB in conjunction with chemotherapy regimens, such as gemcitabine/albumin-bound paclitaxel, there was no observed improvement in the survival duration of the subjects (84). Krishnan K. Mahadevan’s team has validated the synergistic effects of cDC1 vaccines and ICB in various preclinical models. When used alone, ICB is easily resisted by PDAC due to the absence of cDC1 antigen presentation in the TME. However, vaccination with cDC1 vaccines resensitizes PDAC to ICB and can even lead to a complete cure. The combined treatment of cDC1 vaccines and ICB can also establish long-term immune memory, aiding in the prevention of tumor recurrence (48). In a phase II study, a similar combination of GVAX and ICB as a neoadjuvant treatment regimen significantly prolonged the survival of patients with PDAC, with a median disease-free survival (DFS) of 33.51 months and a 49% reduction in the risk of disease recurrence (85). The synergy of a personalized RNA vaccine targeting KRAS G12D and ICB also yields a better effect (86). Moreover, various other immunotherapy combinations, such as oncolytic viruses with ICB, and NKT cell activation therapy with PD-L1 inhibitors, have been proposed and tested, yielding promising results. Among these, CAR-engineered NKT cells co-expressing IL-15 have demonstrated enhanced tumor regression in preclinical PDAC models (57). 41BB, a T-cell co-stimulatory immune checkpoint molecule, promotes T-cell proliferation and enhances T-cell functionality. LAG3, conversely, is an immune checkpoint receptor that can result in the suppression of T-cell functions. In PDAC mouse models, simultaneous administration of 41BB agonists and LAG3 antagonists improves survival and enhances antitumor immunity compared with monotherapy. On the foundation of this dual-agent combination, the addition of a CXCR1/2 inhibitor, which forms a triple therapy, not only reduces tumor size but also demonstrates significant and sustained long-term efficacy (87) (for details, see legend to **Table 2**). Collectively, these representative strategies illustrate how distinct immune barriers can be mapped onto the 3D+R conceptual framework, integrating stromal remodeling, metabolic checkpoint inhibition, antigen priming, and rational sequencing into a unified therapeutic logic (**Table 1**). These approaches exemplify the “Rational sequencing” dimension of the 3D+R framework, demonstrating that barrier-specific interventions must be temporally and biologically coordinated rather than empirically combined.

**Table 2:** Representative clinical outcomes of monotherapy versus combinatorial immunotherapy in PDAC.

No.	Immunotherapy strategy	Monotherapy ORR (%)	Combination ORR/outcome	Reference
1	PD-1 monotherapy (MSI-H PDAC)	18-20%	18-20% (same as monotherapy)	(81)
2	PD-L1 + CTLA-4 dual blockade	PD-L1 alone: <3%	3.1%	(83)
3	PD-1 + CTLA-4 + chemotherapy (Gem/nab-P)	PD-1 + CTLA-4: 3.1%	No improvement	(84)
4	GVAX vaccine + ICB (PD-1 or CTLA-4)	GVAX alone: ~5%	DFS 33.5 mo, recurrence ↓ 49%	(85)
5	cDC1 vaccine + ICB (pre-clinical)	ICB alone: 0% in model	Complete cure in mice	(48)
6	iNKT-IL-15-CAR + PD-L1 inhibitor (pre-clinical)	PD-L1 inhibitor alone: ~5% (est.)	Tumor regression in mice	(57)

Gem/nab-P, gemcitabine plus nab-paclitaxel.

## 11 Summary

Despite incremental systemic gains, PDAC remains largely refractory to immune checkpoint blockade due to a multilayered immunosuppressive TME. Growing evidence highlights the need for framework-driven synthesis that connects mechanistic insight with clinically actionable stratification and rational combination design (88). Here, we conceptualize PDAC as a multidimensional immune ecosystem requiring coordinated therapeutic intervention.

**Figure 3:**
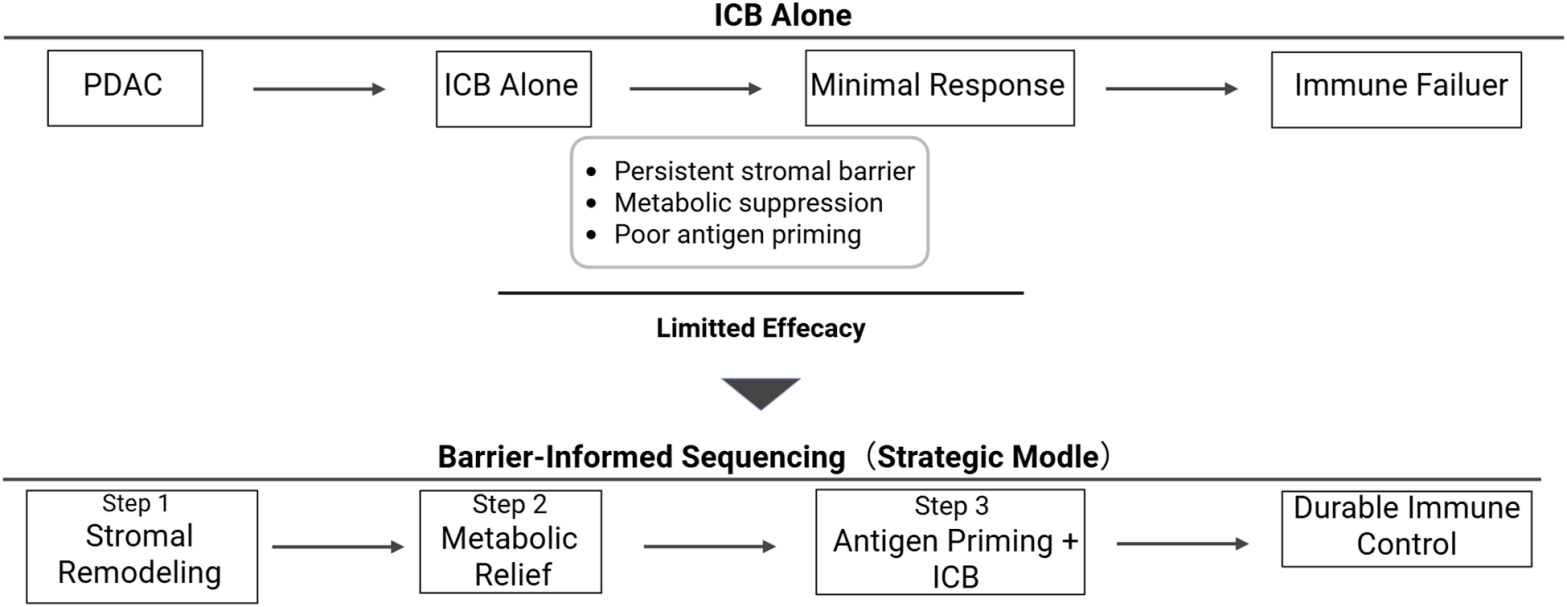
The 3D+R framework for rational immunotherapy in PDAC. Effective immune restoration requires coordinated de-desmoplasia, de-adenosine, and *de-novo* antigen priming, integrated through rational sequencing to overcome multidimensional immune barriers. .

We therefore propose the “3D+R” framework, De-desmoplasia, De-adenosine, *De-novo* antigen, and Rational sequencing, to guide next-generation immunotherapeutic strategies. This model emphasizes coordinated barrier dismantling, biomarker-guided patient stratification, and temporally optimized therapeutic sequencing. A clinical application of this framework may involve tailoring initial intervention according to stromal density, metabolic suppression, and immune phenotype before introducing checkpoint blockade. The framework is schematically summarized in **Figure 3**. Prospective validation of biomarker-defined 3D+R stratification will be necessary to determine its clinical utility. Barrier-informed and temporally coordinated strategies may be necessary to improve the durability of clinical responses in PDAC.
